# Impact of nonalcoholic fatty liver disease on atrial fibrillation recurrence after catheter ablation: a prospective cohort study

**DOI:** 10.3389/fcvm.2026.1747145

**Published:** 2026-03-13

**Authors:** Zhe Xu, Tao Yi, Xuehai Chen, Kezeng Gong, Feilong Zhang, Yunling Lin

**Affiliations:** 1 Department of Cardiology, Fujian Medical University Union Hospital, Fuzhou, China; 2 Fujian Cardiovascular Medical Center, Fuzhou, China; 3 Fujian Institute of Coronary Heart Disease, Fuzhou, China; 4 Fujian Cardiovascular Research Center, Fuzhou, China; 5 Fujian Medical University Heart Center, Fuzhou, China

**Keywords:** atrial fibrillation (AF), catheter ablation, fibrosis, NAFLD, nonalcoholic fatty liver disease

## Abstract

**Background:**

Nonalcoholic fatty liver disease (NAFLD), prevalent in 25% globally, correlates with heightened cardiovascular risks. Retrospective studies suggest NAFLD increases post-ablation atrial arrhythmia recurrence, but prospective evidence is lacking.

**Methods:**

This registered cohort study (ChiCTR2200059079) enrolled 106 AF ablation patients (53 NAFLD vs. 53 non-NAFLD). AF recurrence risk was assessed using multivariable Cox proportional hazards regression. NAFLD was diagnosed per American Association for the Study of Liver Diseases criteria, with fibrosis staged by FIB-4 (Fibrosis-4 index).

**Results:**

NAFLD patients exhibited higher recurrence rates (49.1% vs. 24.5%, *p* = 0.022; annualized incidence: 48.1 vs. 24.0 per 100 person-years). Advanced fibrosis (FIB-4 > 1.30) amplified recurrence risk (71.4% vs. 34.4%, *p* = 0.024). Metabolic indices (BMI/glucose/lipids) did not independently predict outcomes.

**Conclusion:**

NAFLD independently predicts AF recurrence post-ablation, with fibrosis severity stratifying risk. This underscores NAFLD management as a potential therapeutic target.

## Background

Nonalcoholic fatty liver disease (NAFLD), a metabolic liver injury closely associated with insulin resistance and genetic predisposition, is the most common chronic liver disease with a global prevalence of approximately 25% (1). NAFLD is strongly linked to metabolic syndrome, type 2 diabetes mellitus, and atherosclerotic cardiovascular diseases. Notably, 40.8% of NAFLD patients do not meet overweight or obesity criteria, yet still exhibit elevated cardiovascular risks (2).

NAFLD is highly prevalent in atrial fibrillation (AF) populations, with studies reporting up to 42.2% of AF patients having concurrent NAFLD (3). Shared risk factors between NAFLD and AF—including diabetes, dyslipidemia, obesity, hypertension, obstructive sleep apnea, and systemic inflammation—contribute to the initiation and progression of AF. A recent meta-analysis (4) incorporating multiple large cohort studies (total 614,673 participants) revealed that NAFLD significantly increases AF incidence even after adjusting for traditional risk factors. Retrospective data further indicate that NAFLD is an independent predictor of post-ablation atrial arrhythmia recurrence, with its predictive value extending beyond conventional risk factors such as BMI, left atrial size, and epicardial fat volume (5). Prospective studies are urgently needed to confirm the impact of NAFLD on AF recurrence after catheter ablation.

## Objectives

This study aims to:

Investigate the influence of NAFLD on arrhythmia recurrence following atrial fibrillation catheter ablation.

Explore the relationship between NAFLD-related liver fibrosis (assessed via FIB-4 score) and post-procedural arrhythmia recurrence.

## Methods

### Study design and population

This single-center prospective cohort study (registered at the Chinese Clinical Trial Registry, ChiCTR2200059079) consecutively enrolled patients diagnosed with paroxysmal or persistent AF undergoing catheter ablation for symptom management at Fujian Medical University Union Hospital (October 2021–June 2022). Inclusion criteria: Symptomatic paroxysmal or persistent AF scheduled for first-time catheter ablation. Exclusion criteria included: age <18 years; prior NAFLD diagnosis or treatment (diet/exercise/medication); active malignancy; connective tissue disease; severe hepatic/renal dysfunction; rheumatic or congenital heart disease; acute myocardial infarction or cardiac surgery within 12 months. Written informed consent was obtained from all participants, and the study protocol was approved by the institutional ethics committee (Approval No. 2022KY061). Patients were stratified into NAFLD and non-NAFLD groups based on diagnostic criteria established by the American Association for the Study of Liver Diseases (imaging/biopsy + exclusion of secondary causes) (6). All enrolled patients with hepatic steatosis would also meet the MASLD criteria. Alcohol consumption was assessed via detailed history (<20 g/day for men, <10 g/day for women) and serum biomarkers (GGT) to exclude alcoholic liver disease. Secondary causes (viral hepatitis, medications) were excluded via serological testing (HBsAg, anti-HCV) and clinical history. CHA₂DS₂-VASc score was calculated to assess stroke risk in AF patients. A Consolidated Standards of Reporting Trials (CONSORT)-style flow diagram detailing patient screening, enrollment, and exclusion is provided in Supplementary Figure S1.

### Data collection

Demographic data (age, sex, smoking status), medical history (hypertension, diabetes, coronary artery disease, stroke), laboratory parameters [fasting plasma glucose (FPG), alanine aminotransferase (ALT), aspartate aminotransferase (AST), total cholesterol (TC), triglycerides (TG)], echocardiographic indices [left atrial diameter (LAD), left ventricular ejection fraction (LVEF), E/E' ratio], CHA_2_DS_2_-VASc and HAS-BLED scores, and post-procedural medications (antiarrhythmic drugs, anticoagulants) were recorded. All echocardiograms were performed by experienced sonographers using a standardized protocol prior to ablation. Measurements were made offline by two independent cardiologists blinded to patient group, with averaged values used for analysis. AF classification (paroxysmal/persistent) followed the guidelines of the European Society of Cardiology (ESC) (7).

### FIB-4 assessment

FIB-4 index was used as a non-invasive surrogate for liver fibrosis staging. Although histological staging ranges from F0 to F4, we adopted a dichotomized cutoff (FIB-4 ≤ 1.30 for no/early fibrosis vs. >1.30 for advanced fibrosis) based on validated thresholds used in prior NAFLD and cardiovascular outcome studies, which enhances clinical applicability and statistical power in a cohort of this size.

Fibrosis-4(FIB-4) index was calculated as: FIB-4 = [AST (IU/L) × age (years)]/[ALT (IU/L)^1/2^ × platelets (×10⁹/L)] (8).

Patients with FIB-4 ≤ 1.30 were classified as no/early fibrosis (F0–F2), while those >1.30 were considered advanced fibrosis (F3–F4) (8, 9).

### Ablation procedures

#### Radiofrequency ablation

Radiofrequency ablation was performed using the CARTO 3 electroanatomic mapping system (Biosense Webster, Johnson & Johnson). For paroxysmal AF: Bilateral pulmonary vein isolation (PVI) was achieved, with procedural endpoint defined as bidirectional conduction block between the pulmonary veins and left atrium. If concomitant atrial tachycardia was observed, additional interventions—including superior vena cava isolation, linear ablation (e.g., roof or mitral isthmus lines), or substrate modification—were performed at the operator's discretion. For persistent AF: Following PVI, operators determined the necessity of further substrate ablation (lines or complex fractionated electrogram ablation). If AF persisted or transitioned to organized atrial tachycardia, direct current cardioversion was administered. All ablation lines and PV isolation were revalidated during sinus rhythm, with touch-up ablation applied if conduction gaps were detected.

#### Cryoballoon ablation

For cryoballoon ablation, pulmonary vein isolation was achieved using a 28-mm cryoballoon (Medtronic) guided by a circular mapping catheter (Achieve, Medtronic). For paroxysmal AF: Bilateral PVI with bidirectional block confirmation was performed. For persistent AF: After PVI, cardioversion was applied if AF persisted. PV isolation durability was reassessed post-cardioversion, and consolidation ablation was conducted if reconnection occurred.

#### Post-procedural management

Antiarrhythmic drugs (AADs): Persistent AF patients received AADs (amiodarone, propafenone, or metoprolol) under physician guidance unless contraindicated. Paroxysmal AF patients did not receive routine AADs. All AADs were discontinued after the blanking period unless symptomatic atrial arrhythmias recurred. Anticoagulation: Patients continued preoperative anticoagulant regimens [non-vitamin K antagonist oral anticoagulants (NOACs) or warfarin with INR 2.0–3.0] for ≥3 months. Post-blanking anticoagulation was adjusted based on CHA_2_DS_2_-VASc scores and bleeding risk.

### Follow-up

The blanking period was set to 3 months, consistent with consensus statements from the 2020 ESC/EACTS Guidelines for the Management of Atrial Fibrillation, which recommend excluding early arrhythmia episodes as they often reflect transient post-ablation inflammation or reversible electrical remodeling rather than true procedural failure. This approach minimizes overestimation of recurrence and aligns with contemporary AF ablation outcome reporting standards. AF recurrence was defined as electrocardiography-documented atrial tachyarrhythmia (AF/atrial flutter/tachycardia) lasting >30 s beyond the 3-month blanking period. Episodes occurring during the blanking period were recorded but not counted as recurrence; however, their presence did not influence post-blanking outcome analysis.All patients completed ≥12 months of follow-up after ablation, and had scheduled clinic visits at 3, 6, and 12 months. Patients who had any symptoms related to AF were asked to immediately complete an additional outpatient visit. Recurrence data were obtained from electrocardiography analysis, and 3-day Holter monitoring.

### Statistical analysis

The sample size was calculated using PASS 11.0 (NCSS, Kaysville, UT, USA) based on: two equal-sized groups, two-sided *α* = 0.05, *β* = 0.10 (power = 90%), and atrial fibrillation recurrence rates of 56% (NAFLD) vs. 21% (non-NAFLD) from prior studies (5). Initial estimation yielded 39 subjects/group. Accounting for 10% clinical and 15% long-term follow-up attrition rates, the final sample size was 106 (53/group). All statistical analyses were performed using SPSS statistical software version 20 (IBM Corporation, Armonk, New York). Continuous variables were expressed as mean ± SD or median (Q1–Q3) and analyzed using Student's t-test or Mann–Whitney U test. Categorical variables were presented as numbers (percentage) and compared via *χ*^2^ test. Multivariable Cox regression included variables with *p* < 0.10 in univariate analysis. Kaplan–Meier curves compared recurrence-free survival. *p* < 0.05 was considered significant. Annualized incidence rates of AF recurrence per 100 person-years were calculated for the overall cohort and by NAFLD status, along with 95% confidence intervals.

## Results

### Baseline characteristics

A total of 106 patients (53 NAFLD vs. 53 non-NAFLD) completed ≥12-month follow-up. The NAFLD group exhibited significantly higher BMI [25.95 (23.79, 27.34) vs. 24.45 (21.26, 25.80) kg/m^2^, *p* < 0.001], FPG [5.4 (4.8, 6.0) vs. 5.0 (4.7, 5.4) mmol/L, *p* = 0.04], ALT [23 (15, 34) vs. 16 (12, 25) U/L, *p* = 0.02], TG [1.70(1.25, 2.30) vs. 1.02 (0.80, 1.35) mmol/L, *p* < 0.001], TC (4.85 ± 1.03 vs. 4.36 ± 0.97 mmol/L, *p* = 0.01), and E/E' ratio [12 (9.9, 14.8) vs. 10 (8.7, 12.45), *p* = 0.02]. Fibrosis staging (FIB-4 ≤ 1.30) was more prevalent in NAFLD vs. non-NAFLD (60.4% vs. 34.0%, *p* = 0.01). No intergroup differences were observed in age, sex, AF type, or comorbidities (Table 1).

**Table 1:** Baseline characteristics.

Parameter	NAFLD (*n* = 53)	Non-NAFLD (*n* = 53)	*p*-value
Demographics			
Age (years)	61.11 ± 12.87	63.02 ± 12.70	0.44
Male	33 (62.3)	36 (67.9)	0.54
BMI (kg/m^2^)	25.95 (23.79–27.34)	24.45 (21.26–25.80)	**<0**.001*
Comorbidities			
Smoking	15 (28.3)	12 (22.6)	0.50
Coronary heart disease	1 (1.9)	0 (0)	1.00
Diabetes mellitus	10 (18.9)	6 (11.3)	0.28
Hypertension	23 (43.4)	17 (32.1)	0.23
Stroke	2 (3.8)	2 (3.8)	1.0
Persistent AF	16 (30.2)	22 (41.5)	0.22
CHA_2_DS_2_-VASc	1 (1, 2)	1 (0, 2)	0.12
HASBLED	0 (0, 1)	0 (0, 1)	0.38
Laboratory values			
ALT (U/L)	23 (15–34)	16 (12–25)	**0**.02*
AST (U/L)	20 (16–26)	20 (15.5–24)	**0**.**75**
TBIL (mmol/L)	10.7 (8.25, 14))	11.4 (9.55, 15.35)	0.21
Cr (mmol/L)	72 (62, 83)	77 (69, 86.5)	0.07
FPG (mmol/L)	5.4 (4.8–6.0)	5.0 (4.7–5.4)	**0**.04*
TG (mmol/L)	1.70 (1.25–2.30)	1.02 (0.80–1.35)	**<0**.001*
TC (mmol/L)	4.85 ± 1.03	4.36 ± 0.97	**0**.01*
LDL-C (mmol/L)	3.12 ± 0.93	2.77 ± 0.95	0.06
Echocardiography			
LVED (mm)	47.98 ± 4.73	47.21 ± 5.44	0.44
LAD (mm)	38.4 (35.5–41.5)	38.6 (33.0–42.3)	0.57
LVMI (g/m^2^)	97.08 ± 18.13	96.84 ± 22.46	0.95
LVEF (%)	66.27 ± 4.24	66.03 ± 8.01	0.85
E/E’ ratio	12.0 (9.9–14.8)	10.0 (8.7–12.45)	**0**.02*
Antiarrhythmic therapy			0.70
Beta-blocker	2 (3.8)	3 (5.7)	
Amiodarone	5 (9.4)	8 (15.1)	
Propafenone	5 (9.4)	3 (5.7)	
Anticoagulation			0.29
Edoxaban	5 (9.4)	8 (15.1)	
Dabigatran	6 (11.3)	3 (5.7)	
Rivaroxaban	42 (79.2)	40 (75.5)	
Warfarin	0 (0)	2 (3.8)	
Radiofrequency ablation	36 (67.9)	41 (77.4)	0.28

NAFLD, nonalcoholic fatty liver disease; AF, atrial fibrillation; BMI, body mass index; ALT, Alanine transaminase; AST, aspartate transaminase; TBIL, total bilirubin; Cr, creatinine; FPG, fasting plasma glucose; TG, triglyceride; TC, total cholesterol; LDL-C, low-density lipoprotein cholesterol; HDL-C, high-density lipoprotein cholesterol; LVED, left ventricular enddiastolic diameter; LAD, left atrial diameter; LVMI, left ventricular mass index; LVEF, left ventricular ejection fraction.

* *p* < 0.05. Continuous data are presented as mean ± SD or median (Q1–Q3), and categorical data were shown as *n* (%).

Bold values indicate statistical significance (*p* < 0.05).

### Recurrence outcomes

All patients underwent successful catheter ablation without complications (e.g., pericardial tamponade, symptomatic stroke, major bleeding, or death). Radiofrequency ablation was performed in 67.9% of the NAFLD group and 77.4% of the non-NAFLD group (*p* = 0.28).

**Figure 1:**
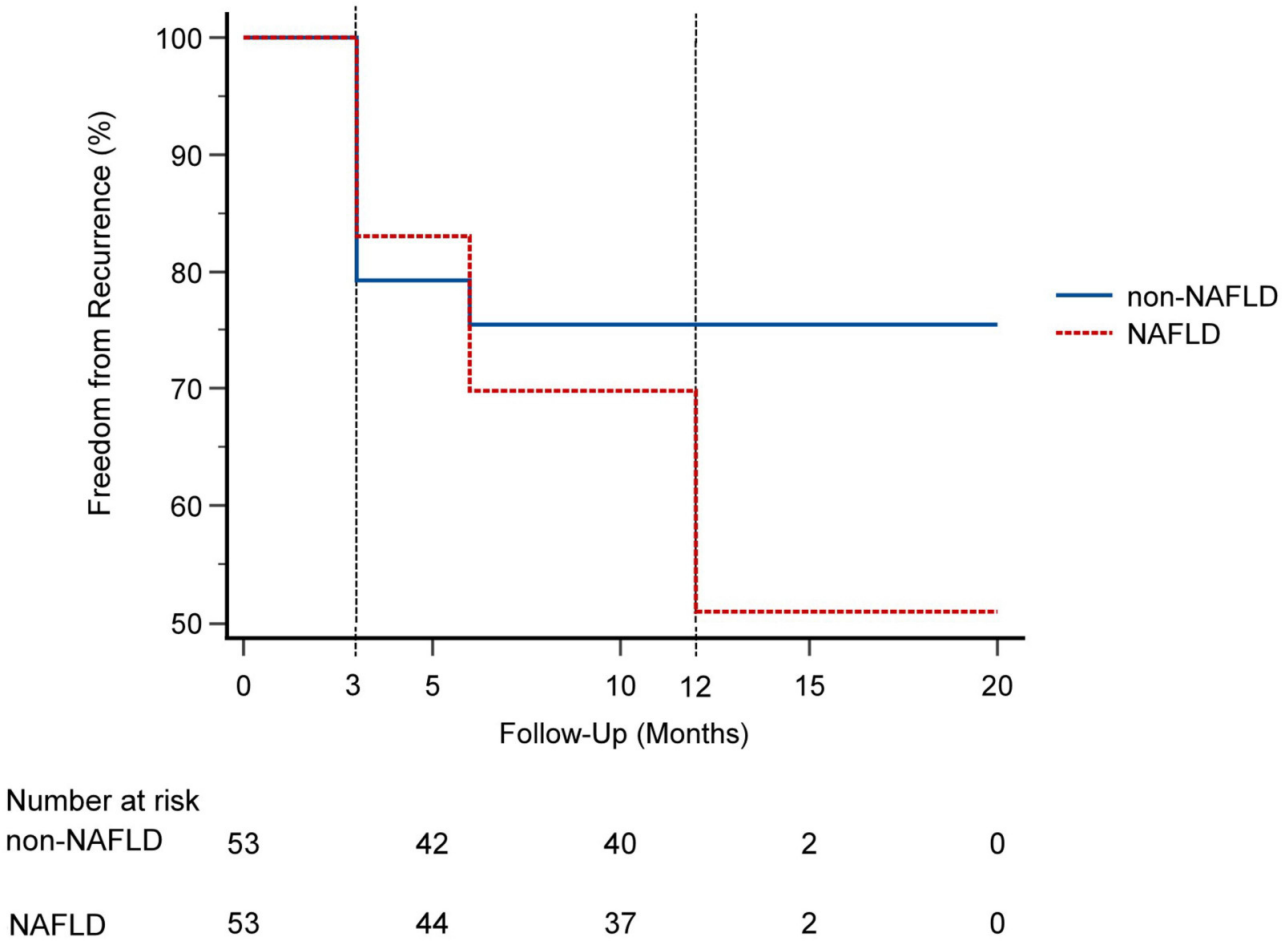
Freedom from atrial fibrillation recurrence after catheter ablation in patients with and without nonalcoholic fatty liver disease (NAFLD). Kaplan–Meier analysis shows a significantly lower probability of remaining free from recurrence in the NAFLD group compared to the non-NAFLD group over a mean follow-up of 12.25 ± 1.29 months (log-rank = 5.212; *p* = 0.022).

**Figure 2:**
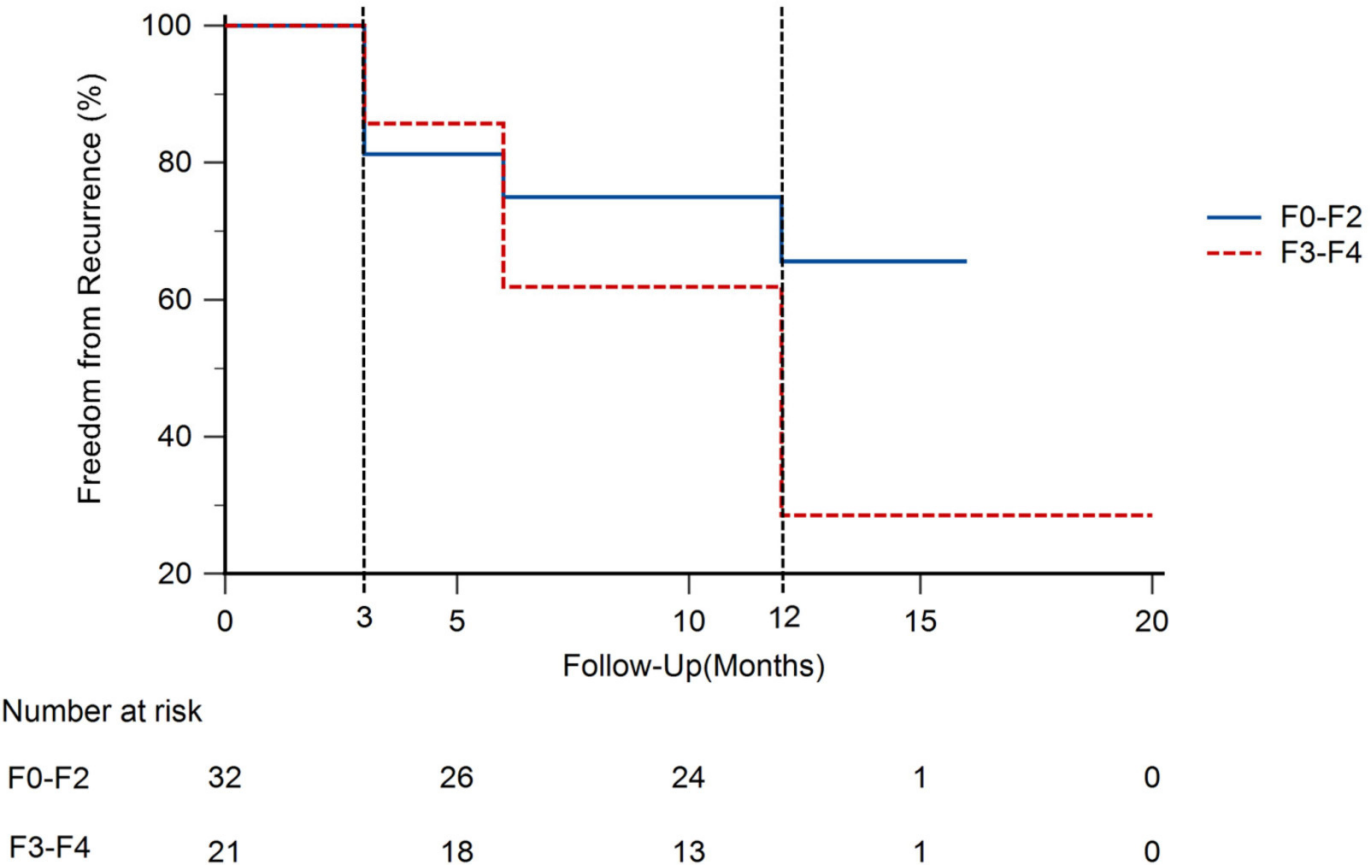
Freedom from atrial fibrillation recurrence after catheter ablation in NAFLD patients, stratified by liver fibrosis stage. Among patients with NAFLD, those with advanced fibrosis (FIB-4 > 1.30, F3–F4) had a significantly lower probability of remaining free from recurrence compared to those with no/early fibrosis (FIB-4 ≤ 1.30, F0–F2) (log-rank = 5.127; *p* = 0.024).

Over a mean follow-up of 12.25 ± 1.29 months, the NAFLD group demonstrated a significantly higher AF recurrence rate compared to the non-NAFLD group(49.1% vs. 24.5%, *p* = 0.022; Figure 1). The annualized incidence of AF recurrence was significantly higher in patients with NAFLD compared to those without (48.1 vs. 24.0 per 100 person-years). The overall annualized incidence was 36.0 per 100 person-years. Within the NAFLD cohort, advanced fibrosis (F3-F4) correlated with increased recurrence (71.4% vs. 34.4%, *p* = 0.024; Figure 2).

### Predictors of recurrence

Univariate Cox analysis identified NAFLD (HR: 2.019, 95%CI: 1.038–3.930, *p* = 0.039), LAD (HR:1.087 per mm, 95%CI: 1.029–1.149, *p* = 0.003), persistent AF (HR:1.864, 95%CI: 1.023–3.748, *p* = 0.042), and NT-proBNP >300 pg/mL (HR:1.978, 95%CI: 1.045–3.748, *p* = 0.036) as predictors. On multivariable adjustment, NAFLD remained independently associated with recurrence (HR: 2.177, 95%CI: 1.100–4.312, *p* = 0.026; Table 2). Results of univariable Cox regression analyses for all tested variables are presented in Supplementary Table S1.

**Table 2:** Predictors of AF recurrence.

Variable	Univariable analysis HR (95% CI)	*p*-value	Multivariable analysis HR (95% CI)	*p*-value
NAFLD	2.019 (1.038–3.930)	**0**.039*	2.177 (1.100–4.312)	**0**.026*
Persistent AF	1.864 (1.023–3.748)	**0**.042*	0.675 (0.318–1.434)	0.307
LAD (per mm increase)	1.087 (1.029–1.149)	**0**.003*	1.053 (0.987–1.123)	0.121
LVMI (per g/m^2^ increase)	1.014 (0.999–1.028)	0.067	1.009 (0.994–1.024)	0.221
NT-proBNP >300 pg/mL	1.978 (1.045–3.748)	**0**.036*	1.288 (0.608–2.725)	0.509

NT-proBNP, N-terminal proB-type natriuretic peptide; other abbreviations as in Table 1.

* *p* < 0.05.

Bold values indicate statistical significance (*p* < 0.05).

## Discussion

In this prospective study, we demonstrated that patients with NAFLD exhibited significantly higher rates of AF recurrence after catheter ablation compared to non-NAFLD counterparts. Furthermore, within the NAFLD cohort, elevated FIB-4 index were independently associated with increased post-ablation recurrence risks, suggesting that both NAFLD presence and hepatic fibrosis severity may serve as prognostic indicators for AF ablation outcomes.

The metabolic triad of obesity, dyslipidemia, and diabetes mellitus, hallmarks of metabolic syndrome, manifested distinctively in our NAFLD population through elevated BMI, TG, TC, and FPG levels compared to controls (*p* < 0.05). This aligns with epidemiological data demonstrating over 60% NAFLD prevalence in obese and diabetic populations (10). While metabolic abnormalities have been extensively linked to AF pathogenesis (11–13) and poorer ablation outcomes (5, 14, 15, 16–19), our multivariable Cox regression analysis revealed no independent association between these parameters (BMI/FPG/TG/TC) and post-ablation recurrence. This intriguing finding suggests potential non-metabolic mechanisms underlying NAFLD-related AF recurrence.This phenomenon could reflect differential mechanistic pathways - where traditional cardiovascular risk factors (e.g., BMI/FPG/TG) primarily mediate AF development in metabolic syndrome, whereas alternative pathways (e.g., hepatic-derived inflammatory mediators) may dominate in NAFLD-related AF progression.

Emerging evidence posits dual-pathway mechanisms connecting NAFLD with AF: metabolic syndrome-associated pathways and direct hepatocardiac interactions. Meta-analyses by Mantovani et al. (20) and Mendelian randomization studies (21) support NAFLD's independent association with AF beyond conventional metabolic risk factors (22–24). Our findings extend this paradigm by identifying structural cardiac remodeling (evidenced by elevated E/e' ratios and left atrial diameter associations) as potential mediators. Notably, the progressive relationship between hepatic fibrosis severity (stratified by FIB-4 index) and AF recurrence risk corroborates Kang et al.'s observations of increased AF incidence in advanced vs. early fibrosis stages (23, 25).

The dramatically increased risk of arrhythmia recurrence in patients with NAFLD may arise from multiple interconnected pathophysiological mechanisms, including epicardial adipose tissue-mediated electromechanical remodeling (26, 27), systemic inflammation-induced structural/electrophysiological alterations (28, 29), and insulin resistance-mediated metabolic cardiomyopathies (30).

These findings carry important clinical implications. Given evidence that weight loss (≥10%) improves AF ablation success rates and glycemic control enhances rhythm control (5, 16, 31), our study underscores the importance of integrating NAFLD screening and management into peri-ablation care protocols. Targeted interventions addressing hepatic steatosis/fibrosis progression might synergistically improve both hepatic and cardiac outcomes.

### Limitations

This study has several limitations. The research was conducted at a single center with a moderate sample size and limited follow-up duration. Our primary endpoint was the first documented AF episode >30 s beyond the 3-month blanking period, which is standard but does not capture AF burden, duration of episodes, or the clinical impact of recurrent events, which may be equally important. Our follow-up protocol relied on scheduled visits and symptom-triggered monitoring. Asymptomatic AF recurrences may have been under-detected, potentially biasing recurrence rates downwards, albeit non-differentially between groups unless symptom perception differs systematically by NAFLD status. Undiagnosed secondary hepatic steatosis could potentially confound the results. Bidirectional interactions between NAFLD and cardiomyopathy remain incompletely elucidated. Additionally, validation of the FIB-4 score in atrial fibrillation-specific NAFLD populations has not been established.

## Conclusion

To our knowledge, this represents the first prospective study delineating NAFLD's independent prognostic value in AF catheter ablation outcomes. Our findings suggest that NAFLD management should be incorporated into peri-procedural care algorithms, particularly through fibrosis staging using validated tools like FIB-4. Further large-scale multicenter studies with extended follow-up durations are warranted to validate these observations and explore mechanotherapeutic targets.

## Data Availability

The raw data supporting the conclusions of this article will be made available by the authors, without undue reservation.
