# Supplementary material for: Impact of nonalcoholic fatty liver disease on atrial fibrillation recurrence after catheter ablation: a prospective cohort study
Source: Front Cardiovasc Med. 2026 Mar 13;13:1747145. doi: 10.3389/fcvm.2026.1747145 (PMC13021415; doi:10.3389/fcvm.2026.1747145)
Supplement: Supplementary file 1 [file Datasheet1.docx]

**Supplementary Table 1. Results of univariable Cox regression analyses for all tested variables**

| Variable | Univariable Analysis HR (95% CI) | *p*-value |
| --- | --- | --- |
| **Demographics** |  |  |
| Age (years) | 0.987(0.962-1.012) | 0.306 |
| Male | 1.085(0.786-1.498) | 0.619 |
| BMI (kg/m²) | 1.023(0.951-1.101) | 0.537 |
| **Comorbidities** |  |  |
| Smoking | 1.005(0.701-1.440) | 0.979 |
| Coronary heart disease | 0.535(0.197-1.450) | 0.219 |
| Diabetes mellitus | 0.792(0.537-1.169) | 0.241 |
| Hypertension | 1.011(0.732-1.397) | 0.945 |
| Stroke | 0.774(0.380-1.577) | 0.480 |
| Persistent AF | 1.864 (1.023-3.748) | **0.042*** |
| CHA_2_DS_2_-VASc | 0.994(0.740-1.335) | 0.968 |
| HASBLED | 1.020(0.7629-1.654) | 0.937 |
| **Laboratory Values** |  |  |
| ALT (U/L) | 0.995(0.980-1.011) | 0.561 |
| AST (U/L) | 1.003 (0.991-1.015) | 0.663 |
| TBIL (mmol/L) | 0.988(0.952-1.025) | 0.517 |
| Cr (mmol/L) | 1.004(0.998, 1.009) | 0.172 |
| FPG (mmol/L) | 1.054 (0.910-1.221) | 0.484 |
| TG (mmol/L) | 0.934(0.693-1.260) | 0.656 |
| TC (mmol/L) | 0.851(0.622-1.166) | 0.315 |
| LDL-C(mmol/L) | 0.929(0.661-1.307) | 0.674 |
| NT-proBNP ( pg/mL) | 1.000 (1.000-1.000) | **0.037*** |
| NT-proBNP >300 pg/mL | 1.978 (1.045-3.748) | **0.036*** |
| **Echocardiography** |  |  |
| LVED(mm) | 1.044(0.982-1.109) | 0.166 |
| LAD (mm) | 1.087(1.029-1.149) | **0.003*** |
| LVMI(g/m²) | 1.014(0.999-1.028) | 0.067 |
| LVEF(%) | 0.978(0.932-1.028) | 0.384 |
| E/E' ratio | 1.004 (0.922-1.093) | 0.932 |
| **NAFLD** | 2.019 (1.038-3.930) | **0.039*** |
| **Radiofrequency ablation** | 0.935(0.465-1.878) | 0.850 |

∗*p* < 0.05. Continuous data are presented as mean±SD or median (Q1–Q3) , and categorical data were shown as n (%).

Abbreviations: NAFLD, nonalcoholic fatty liver disease; AF, atrial fibrillation; BMI, body mass index; ALT, Alanine transaminase; AST, aspartate transaminase; TBIL, total bilirubin; Cr, creatinine; FPG, fasting plasma glucose; TG, triglyceride; TC, total cholesterol; LDL-C, low-density lipoprotein cholesterol; HDL-C, high-density lipoprotein cholesterol; LVED, left ventricular enddiastolic diameter; LAD, left atrial diameter; LVMI,left ventricular mass index; LVEF, left ventricular ejection fraction.


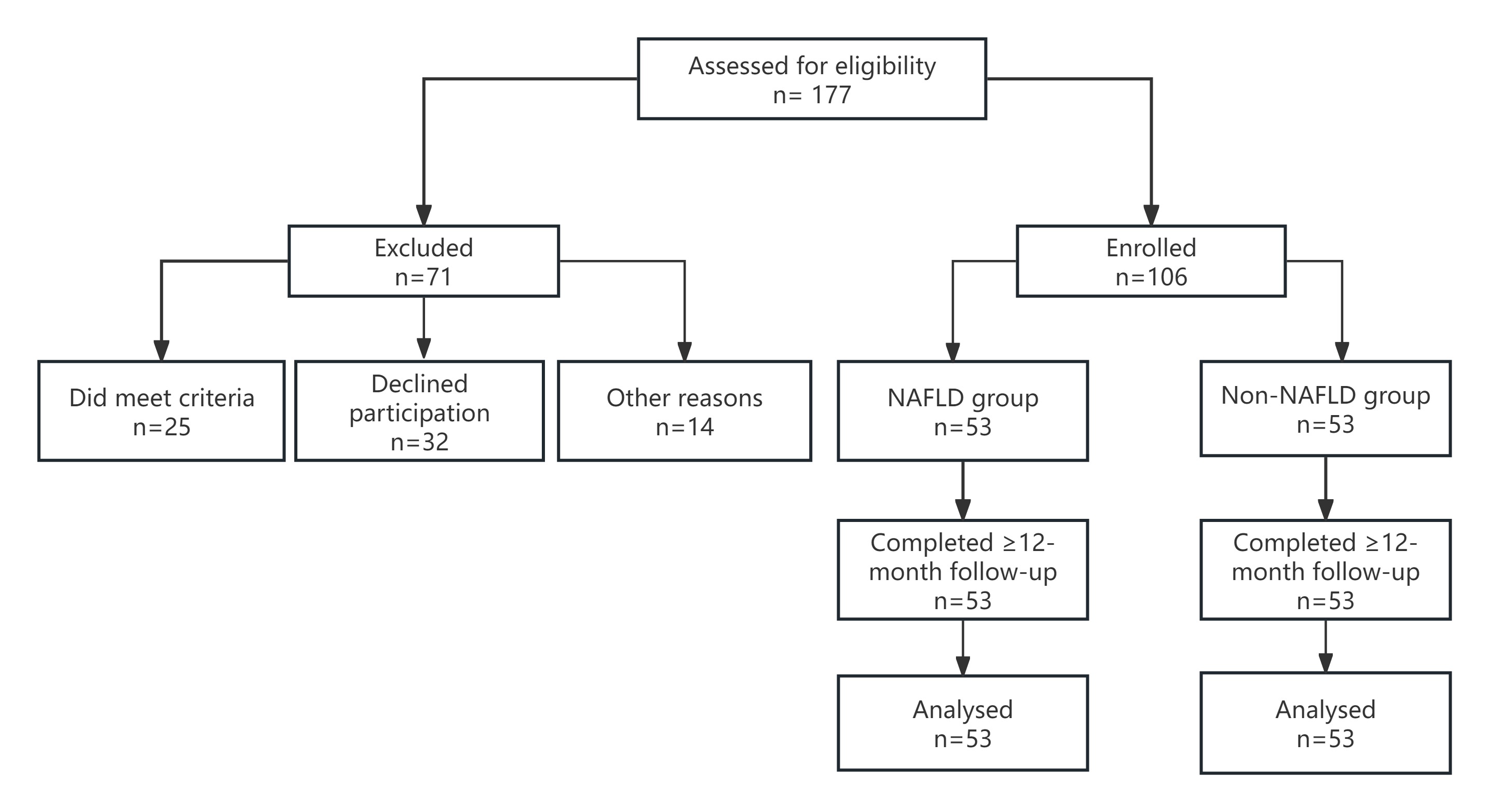


**Supplementary Figure 1.** Patient screening, enrollment, and follow-up flowchart.
This CONSORT (Consolidated Standards of Reporting Trials)-style diagram details the flow of participants through the prospective cohort study. A total of 177 consecutive patients with atrial fibrillation (AF) scheduled for catheter ablation were assessed for eligibility. Following the application of exclusion criteria, 106 patients were enrolled and stratified into nonalcoholic fatty liver disease (NAFLD) $(n=53$) and non‑NAFLD $(n=53$) groups. All enrolled patients underwent successful ablation and completed ≥12 months of follow‑up, with none lost to analysis. The specific reasons for exclusion are listed.
